# Single coracoclavicular suture fixation with Mersilene tape versus hook plate in the treatment of acute type V acromioclavicular dislocation: a retrospective analysis

**DOI:** 10.1186/s13018-018-0831-0

**Published:** 2018-05-16

**Authors:** Ying-Cheng Huang, Shan-Wei Yang, Chun-Yu Chen, Kai-Cheng Lin, Jenn-Huei Renn

**Affiliations:** Department of Orthopedics, Kaohsiung Veterans General Hospital, No. 386, Ta-Chung 1st Rd, Kaohsiung, 81346 Taiwan, Republic of China

**Keywords:** Acromioclavicular dislocation, Coracoclavicular fixation, Mersilene tape, Loop suspensory fixation, Hook plate

## Abstract

**Background:**

Here, we compared the clinical and radiographic outcomes between coracoclavicular (CC) fixation with Mersilene tape and hook plate for acute unstable acromioclavicular (AC) joint dislocation treatment.

**Methods:**

We enrolled 49 patients with unstable acute AC dislocation who, between January 2010 and January 2014, underwent surgery with single CC suture fixation with Mersilene tape (M group, 25 cases) or clavicle hook plate (H group, 24 cases). In M and H groups, the average age was 43.7 (range 18–72) and 42.0 (range 17–84) years, the male to female ratio of each group was 15:20 and 19:5, and the injured side left to right ratio was 12:13 and 11:13, respectively. All patients were right-handed. We retrospectively compared the operation time, complication rate, visual analog scale (VAS), University of California at Los Angeles (UCLA) shoulder rating scale, Oxford shoulder scores, and the radiographic outcomes based on reduction loss of CC distance on postoperative follow-up.

**Results:**

No significant difference in patient demographics between the two groups in age (*p* = 0.709), gender (*p* = 0.217), time from injury to surgery (*p* = 0.863), and injured side (*p* = 1.000). The mean follow-up was 26.2 months (range 24–35 months). Nine cases of reduction loss (36%) and one of distal clavicle osteolysis (4%) were noted in the M group. CC distance improvement in the H group was significantly superior to that in the M group at 3 months (before hook plate removal, *p* < 0.001) and 12 months postoperatively (after hook plate removal, *p* = 0.004), while subacromial erosions were revealed in nine cases (37.5%) in the H group. No significant difference in operative time (*p* = 0.846), complication rate (*p* = 1.000), VAS (*p* = 0.199), mean UCLA shoulder rating scale (*p* = 0.353), and Oxford shoulder (*p* = 0.224) scores between the two groups.

**Conclusions:**

Both hook plate and Mersilene tape fixations provided temporary stabilization of acute type V AC dislocation and yielded comparable clinical outcomes. The hook plate provided better maintenance of reduction of radiographic outcomes. CC suture fixation with Mersilene tape may serve as an alternative method of stabilization which provides acceptable outcome without the need of implant removal.

## Background

Acromioclavicular (AC) joint dislocations are relatively common injuries in young athletes. These account for over half of all sports-related shoulder injuries, particularly in men [1, 2]. According to the Rockwood classification system, conservative treatment is acceptable for Rockwood type I and II AC joint dislocations. However, the surgical intervention methods indicated for type III, IV, V, and VI AC joint dislocations continue to remain a matter of debate [3–6].

Thus far, many surgical interventions have been developed for the management of displaced AC dislocation, e.g., AC or coracoclavicular (CC) joint reconstruction with or without tendon transfer, CC fixation with screws and sutures, Weaver–Dunn procedure, arthroscopic-assisted surgery, and loop suspensory fixation [7–14]. However, there is no current consensus regarding the most suitable method.

In recent years, hook plate fixation and CC fixation using a wide loop suspensory material have come to be widely used treatment methods [15, 16]. Hook plates serve as an internal fixation which contributes to fracture healing in distal clavicle fractures and ligament scarring in AC dislocations. With the strong holding fixator in the bony part, hook plates allow early mobilization. Implant removal tends to be the main complaint of patients who undergo hook plate fixation [17]. In contrast, the loop suspensory fixation for CC restoration reduces patient discomfort, as it does not require a secondary surgery for implant removal, and there is no risk of metal migration, increased stress, or implant failure, particularly in patients with severe osteoporosis [18].

The aim of this study was to compare the clinical and radiographic outcomes between CC suture fixations with loop suspensory fixation using Mersilene tape and hook plate fixations for the treatment of acute unstable AC joint dislocation. The hypothesis of our study is CC fixation with Mersilene tape provides the immediate stabilization and function outcome similarly as hook plate in the treatment of acute type V AC dislocation.

## Methods

**Fig. 1:**
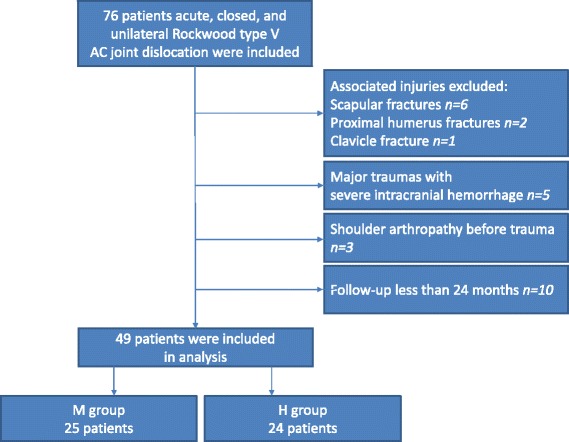
Flowchart of inclusion and exclusion of participants in current analysis

Two experienced orthopedic trauma surgeons treated the patients and randomly decided on the surgical method according to personal experience. Between February 2010 and November 2014, we retrospectively reviewed 76 consecutive patients with Rockwood type V AC joint dislocation undergoing surgical interventions. We included adults with acute trauma injury, closed, and unilateral AC joint dislocation who planned to undergo open reduction and internal fixation by single CC suture fixation with Mersilene tape (Ethicon, Somerville, NJ, USA) or clavicle hook plate (Synthes-Stratec Medical, Solothurn, Switzerland). Patient exclusion criteria were as follows: (1) associated fracture of the clavicle, coracoid process, glenoid, acromion, scapula wing, or proximal humerus; (2) associated brain injury with peripheral neuropathy or cognition impairment who cannot complete the functional score; (3) abnormal shoulder function before injury; or (4) less than 2-year follow-up. Finally, 49 patients were included in this study (Fig. 1). All patients were victims of motorcycle accident. The institutional review board of Kaohsiung General Veterans Hospital approved this retrospective study, and informed consents were taken from all the patients.

**Fig. 2:**
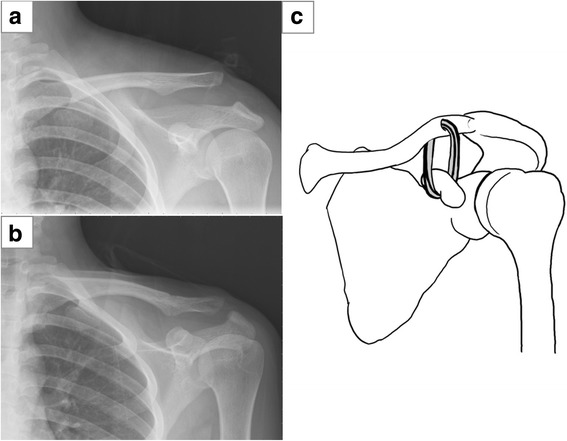
Coracoclavicular (CC) suture fixation with Mersilene tape for a type V AC dislocation after motorcycle accident injury. **a** Preoperative plain radiograph. **b** 1-month postoperative plain radiograph. **c** Surgical technique illustration of suspensory sling fixation with Mersilene tape

**Fig. 3:**
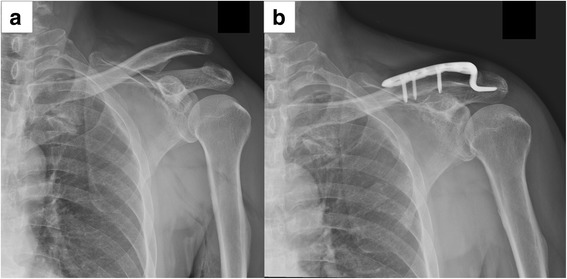
Coracoclavicular fixation with hook plate for a type V AC dislocation after motorcycle accident injury. **a** Preoperative plain radiograph. **b** 1-month postoperative plain radiograph

According to the surgical technique, 25 patients underwent the CC fixation with Mersilene tape (M group) (Fig. 2), and 24 patients underwent open reduction and internal fixation with clavicle hook plate (H group) (Fig. 3).

In the M and H groups, the average age was 43.7 (range 18–72) and 42.0 (range 17–84) years, the male to female ratio of each group was 15:20 and 19:5, and the injured side left to right ratio was 12:13 and 11:13, respectively. All patients were right-handed.

For the M group, surgery was performed under general anesthesia, with the patient in supine position, with the head elevated at 45° and the injured limb freely mobile. A 6-cm longitudinal skin incision was made in line from the clavicle to the coracoid process. Preparation for clavicle tunnel placement was performed first by drilling a 2.5-mm hole slightly anterior to the mid-portion of the distal clavicle at the insertion point of the CC ligament (roughly 2 cm medial to the distal end). To access the base of the coracoid process, a right-angle dissector was used to pass the Mersilene tape through the inferior base of the coracoid process and through the prepared clavicle tunnel. We pressed the clavicle down until it was solidly over-reduced to the original AC anatomic position. The Mersilene tape was passed through the clavicle tunnel and then tied for CC stabilization. The AC joint capsule and the surrounding deep deltotrapezial fascia were repaired with 1–0 Vicryl (Ethicon, Cornelia, GA, USA) direct suture for augmentation.

For the H group, a linear incision parallel to the distal clavicle was made. After the AC joint was exposed and reduced, and the subacromial space was confirmed, the hook portion of the plate was inserted under the acromion. The clavicle portion of the plate was contoured and fixed with screws.

Postoperative treatment following the surgical procedure in both groups was standardized and included a consecutive rehabilitation program. Patients were required to wear an arm sling during 4 weeks to protect the injured shoulder, and early postoperative shoulder mobilization was encouraged. The range of motion of injured shoulder might be limited in the first week due to wound pain, and patients were instructed to perform pendulum exercises after the wound condition and the pain severity improved. In the H group, all hook plates were removed 4 months postoperatively.

**Fig. 4:**
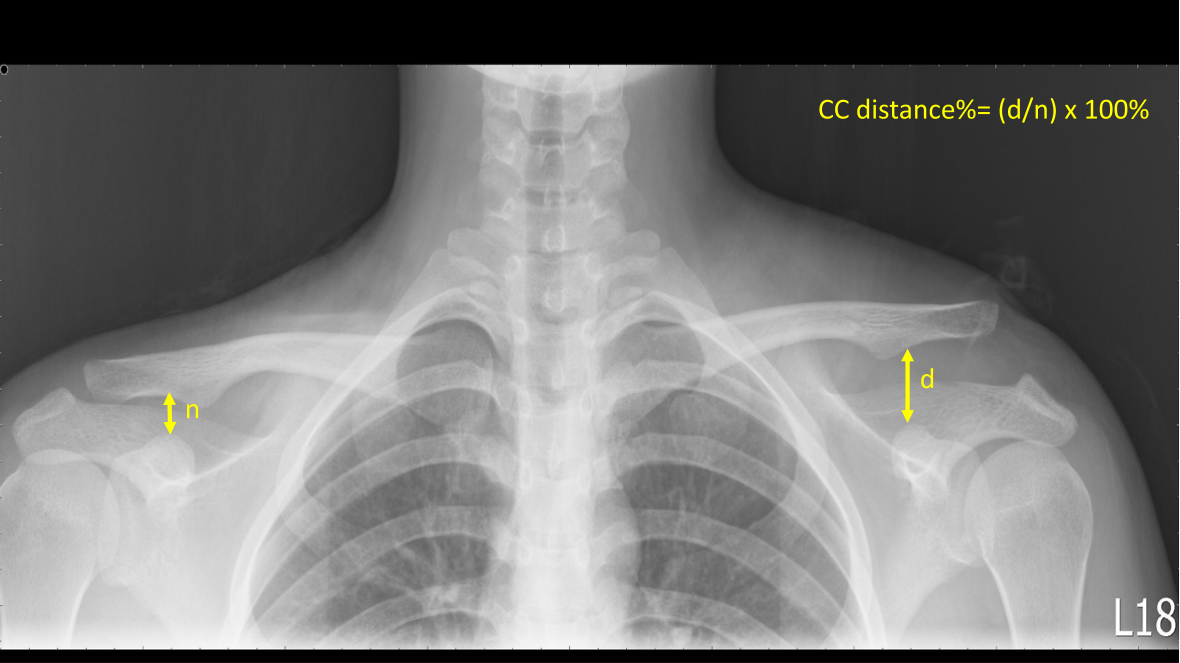
The method of measurement for CC distance

Consecutively, comparative plain radiographs were obtained at the time of initial trauma and at 1, 3, 6, and 12 months of postoperative follow-up. The images were analyzed and standardized to assess the CC distance (height in percent to the contralateral shoulder between the upper border of the coracoid process and the inferior cortex of the clavicle, Fig. 4). We considered increases in CC distance on the final follow-up radiographs of 0–50%, 50–100%, and > 100% with respect to the contralateral side as mild reduction loss, subluxation, and redislocation, respectively [19]. Additionally, complications were evaluated in radiographic images and clinical symptoms by postoperative follow-up.

The clinical outcome evaluations were assessed at 24 months and included (1) the visual analogue scale (VAS) for pain and satisfaction score (0–10), (2) the clinician-completed University of California at Los Angles (UCLA) shoulder rating scale [20], which was stratified by good to excellent result (27–35 points) or fair to poor result (< 27 points), and (3) the patient-completed Oxford shoulder score [21], which was stratified into satisfactory function (40–48 points), mild to moderate dysfunction (30–39 points), moderate to severe dysfunction (20–29 points), or severe dysfunction (0–19 points).

The radiographic and clinical results were followed and reviewed by one independent observer. Statistical analyses were conducted using the Statistical Package for the Social Sciences (SPSS) version 22.0 (SPSS Inc., Chicago, USA). Data were analyzed using Shapiro–Wilk test to explore normality of the tested variables for the small case number of each group. Independent Student’s *t* test or Mann–Whitney *U* test was used to compare continuous variables for normal or abnormal distribution, respectively. Fisher exact test was used to compare the categorical variables. Paired Student’s *t* test was used to compare the difference between before and after the surgery. Significant difference across the two groups was set at *p* < 0.05. Values are given as mean ± standard deviation.

## Results

**Fig. 5:**
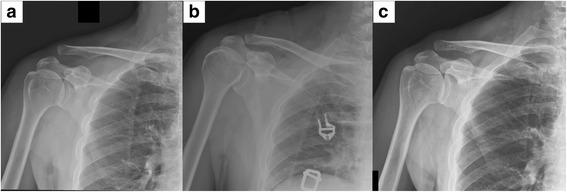
Reduction loss after coracoclavicular fixation with Mersilene tape. **a** Preoperative plain radiograph. **b** 1-month postoperative follow-up. **c** 6-month postoperative follow-up with reduction failure

**Fig. 6:**
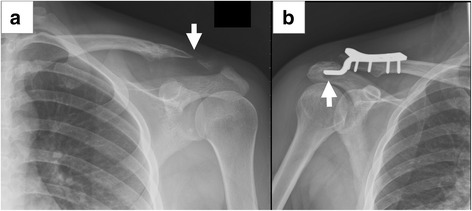
**a** Osteolysis was observed on the left distal clavicle after CC fixation with Mersilene tape (arrow). **b** Subacromial erosion at the stress concentration area (arrow) after CC fixation with hook plate

There was no significant difference in patient and clinical characteristics between the two groups in age (*p* = 0.709), gender (*p* = 0.217), time from injury to surgery (*p* = 0.863), and injured side (*p* = 1.000) (Table 1). The mean follow-up was 26.2 months (range 24–35 months). Regarding the operation time, there was no significant difference between the M and H groups (*p* = 0.846). As for the complication rate, it was 40% (10 cases) in the M group and 37.5% (9 cases) in the H group, without significant difference (*p* = 1.000). Such complications were analyzed in detail. In the M group, nine cases of reduction loss (Fig. 5). One case of distal clavicle osteolysis (Fig. 6a) was identified in the M group, and complete examinations and history taking revealed no secondary intervention, infection, trauma, shock wave treatment, or heavy work over the diseased limb. The possible reason for the osteolysis is the stress concentration over the clavicle site after the CC fixation with Mersilene tape. However, the patient did not have any complaint on her shoulder. In the H group, nine cases of subacromial erosion were identified (Fig. 6b). In our study, the subacromial erosion was defined as a complication, which was identified in 37.5% patients in the H group. Subacromial impingement and the limited motion were the main complaints but the symptoms improved after removal of the hook plate.

**Table 1:** Patient demographics

Parameter	M group	H group	*p* value
Number of patients	25	24	
Age (years)	43.7 ± 15.6	42.0 ± 14.9	0.709^a^
Gender (M/F)	15/20	19/5	0.217^b^
Left/right	12/13	11/13	1.000^b^
Time from injury to surgery (days)	6.00 ± 8.35	8.54 ± 12.61	0.863^c^

Data are expressed as mean ± standard deviation

^a^Independent *t* test

^b^Fisher exact test

^c^Mann–Whitney *U* test

**Fig. 7:**
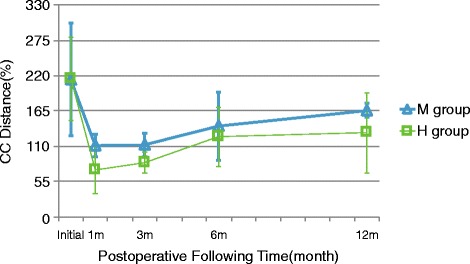
Radiographic outcomes. The line chart illustrated the trend of CC distance of the M and H groups by postoperative follow-up time

CC distances were evaluated consecutively and recorded for statistical analysis (Fig. 7). At the initial trauma, the mean CC distance was not significantly different between the M and H groups (213.8 ± 87.92% vs 214.99 ± 64.43%, *p* = 0.490). After surgery, the mean CC distance was improved to 111.37 ± 17.34% in the M group and 73.77 ± 37.12% in the H group at 1 month postoperatively. The mean CC distance was significantly improved in both groups (*p* < 0.001) after operation.

There were nine patients in the M group with reduction loss at 3 months postoperatively, and the mean CC distance in the M group was 112.08 ± 18.15%, which was significantly greater compared with that in the H group at 84.55 ± 16.84% (*p* < 0.001). At 6 months postoperatively, the CC distance increased in both groups (141.05 ± 53.10% in the M group and 124.72 ± 45.87% in the H group) without significant difference (*p* = 0.126). At 12 months postoperatively, the CC distance increased continuously in both groups, and a significant difference was noted between the H (130.96 ± 62.72%, range 53.3–370.0%) and M groups (166.02 ± 10.61%, range 56.0–270.0%) (*p* = 0.004). There was a high standard deviation in the H group. In our study, we removed the implant for the patients in the H group at 4 months postoperatively. The CC distance of certain patients in the H group increased at the 6 months postoperatively, while the maintenance of CC distance after implant removal in the other patients of the H group were still observed. At 12 months postoperatively, the CC distance of some patients after implant removal increased more, which might be the reason of high standard deviation in the H group. During the postoperative follow-up, the CC distance was lost gradually in both groups. However, the H group presented better maintenance of reduction than the M group.

Clinical outcome measures were assessed and demonstrated in Table 2, and there was no significant difference between the M and H groups in neither evaluation.

**Table 2:** Clinical outcomes in both groups

Parameter	M group	H group	*p* value
Operation time (min)	91.60 ± 13.21	90.63 ± 20.71	0.846^a^
Complication rate	*n* = 10/25 (40%)	*n* = 9/24 (37.5%)	1.000^b^
VAS	1.17 (1–2)	1.36 (1–3)	0.199^c^
UCLA scale	33.3 (28–35)	33.0 (29–35)	0.353^c^
Oxford score	12.8 (12–16)	12.4 (12–15)	0.224^c^

Data are expressed as mean ± standard deviation, ratio (percentage), or mean (range)

^a^Independent *t* test

^b^Fisher exact test

^c^Mann–Whitney *U* test

## Discussion

The methods to treat acute Rockwood type V AC joint dislocation have been discussed extensively. Thus far, arthroscopic-assisted CC reconstruction or CC reconstruction with autologous hamstrings repair, CC fixation with suture anchor or triple button device, and AC fixation with Knowles pin or hook plate for AC joint dislocation have been reported. However, a standardized procedure has yet to be established owing to various complications such as metal breakage, implant loosening, recurrence of instability, metal migration, and neurovascular injury [22–25].

Arirachakaran et al. conducted a systematic review and meta-analysis in which they concluded that the loop suspensory fixation of the CC joint had a higher postoperative functional Constant–Murley score when compared with hook plate for the treatment of acute high-grade AC dislocation [26]. However, long-term follow-up to determine the possibility of chronic rotator cuff degeneration and arthropathy is needed. With sonographic follow-up evaluation, Lin et al. claimed that clavicle hook plate may induce subacromial shoulder impingement and rotator cuff lesion [27].

Clavicle hook plate is widely used because of its secure fixation against rotational, horizontal, and vertical forces. Additionally, it provides satisfactory clinical results [28, 29]. However, this method may result in subacromial erosions, which may induce an increase in these patients’ vulnerability to complications, including distal clavicle fracture, scapular fracture, rotator cuff arthropathy, or secondary arthritis [30, 31]. In case of secondary reduction loss, Baets et al. [32] and Faraj and Ketzer [33] do not even recommend routine implant removal. However, to avoid complications such as secondary hardware breakage, clavicle fracture, imminent subacromial osteolysis, rotator cuff injury, or impingement syndromes, ElMaraghy et al. still recommended removal of the hook plate [34]. In our study, the subacromial erosion was identified in 37.5% patients in the H group, which may be attributed to the stress-rising effect as patients’ shoulder abduction, and the limited motion and subacromial impingement symptoms improved after removal of the hook plate.

Some researchers have reported that AC reconstruction with ligament achieved the anatomic and biomechanical perspectives [35, 36]. However, with such reconstruction, donor site comorbidity must be considered. Currently, several CC suture fixation materials have been developed for AC dislocation, e.g., button suspensory TightRope® (Arthrex, Naples, USA), suture augmentation with absorbable polydioxanonesulfate (PDS) sling, and artificial ligaments (LIGASTIC; Orthomed, Nice, France) [16, 23, 25]. CC suture fixation with Mersilene tape was first proposed by Yang et al. for the reduction of distal clavicle fracture [37]. Chen et al. reported the same clinical outcomes with Mersilene tape and clavicle hook plate for distal clavicle fracture treatment [38]; we applied a similar surgical technique to ours for treating AC dislocation [15].

In our study, we compared CC suture fixation with Mersilene tape and CC fixation with hook plate for the treatment of type V AC joint dislocation. The results indicated that both methods can provide temporary reduction for acute AC joint dislocation. However, the presented complication rate was really high. The complication criteria of our study included subacromial erosion, loss of CC distance, and osteolysis, even without symptoms. The increase of CC distance in the H group was possibly attributed to the implant removal at 4 months postoperatively; however, the CC distance in the M group increased gradually without any trauma episode. In the short term, the radiographic result of 12 monthly follow-ups in our study indicated there was a better reduction using hook plate for acute AC dislocation. However, the nature of movable joint as AC joint allowed patients to perform the shoulder range of motion after implant removal, which may cause certain patients with subluxation with incomplete healing of the AC joint. In the long term, the functional result of 24 monthly follow-ups in our study demonstrated there was no significant difference between the H group and M group. In our cohort of H group, the patients had to undergo removal of implant 4 months postoperatively with a view to avoidance of severe subacromial erosion, shoulder arthritis, and limited abduction of disease shoulder. We focused on the treatment of acute type V AC dislocation, including the immediate reduction and pain relief, that is, reducing the CC distance. The functional score showed there was no significant difference between the H group and M group at 24 months postoperatively. Even though those patients treated with Mersilene tape were found with relatively poor maintenance reduction in radiographic follow-up, they presented similar satisfactory clinical results to those undergoing hook plate fixations. Thus, Mersilene tape remained an option for the treatment of type V dislocation in the acute trauma.

In our study, all the hook plates were removed 4 months postoperatively. Although the hook plate provided greater strength to maintain the reduction than the Mersilene tape, gradual increasing CC distance was also observed after removal of the hook plate. The result demonstrated the H group achieved the significantly superior reduction just after the surgery, though both groups were found with gradual reduction loss on the consecutive follow-up plain films. The holding power of the Mersilene tape seems not strong enough to maintain the anatomic reduction as the hook plate.

Regarding the CC fixation material, Eschler et al. reported that failure of anatomic correction when using PDS for CC fixation resulted in 12% (3 cases of 25) redisplacement (100% increase in CC distance) and 16% (4 cases of 25) of partial reduction loss [16]. They concluded that the hook plate restores the CC distance more accurately than augmentation with a PDS sling, without any difference in the final functional results, even though there were a few cases of hook plate with initial overcorrection and acromial osteolysis in follow-up radiographs. In our study, we experienced similar results. We performed the VAS, UCLA scale, and Oxford score evaluations at 24 months postoperatively, and our results showed no significant difference in pain, satisfaction, or specific restriction of activities of daily life between the two groups. However, CC suture fixation with Mersilene tape led to one case of distal clavicle osteolysis, which might be attributed to the stress effect concentrated on the contact surface of the distal clavicle with the suspensory sling technique; however, our patients did not complain of any discomfort during outpatient follow-up. In contrast, hook plates provide a better reduction, but more subacromial erosions, which resulted in shoulder discomfort. The main complaint with subacromial erosion in the H group was the limited range of abduction of diseased shoulder. In our study, such symptoms subsided after implant removal. Yoon et al. compared the result of patient with and without subacromial erosions using hook plate for the treatment of AC dislocation, and the analysis showed no significant differences in the VAS score or Constant–Murley score at the final follow-up between patients with and without erosion. In radiographic finding, there was no difference in initial CC distance and final CC distance [22].

Besides, patients treated with hook plate require a second intervention for implant removal, that is, another hospitalization and further medical expenditures are needed. By comparison, CC fixation using Mersilene tape offered immediate stabilization of AC dislocation without the need for another implant removal surgery.

This study had several limitations, including the limited number of presenting cases, the non-randomized retrospective study design, and the lack of evaluation for AC joint horizontal instability. Longer follow-up to observe postoperative rotator cuff arthropathy and AC arthropathy is required.

## Conclusions

From this study, surgical techniques using either Mersilene tape or hook plate could provide temporary stability of the AC joint, but the presented complication rate of both methods was really high (40 and 37.5%). The maintenance of reduction using hook plate was significantly superior to that achieved using Mersilene tape, even though there was no significant difference in VAS, UCLA scale, and Oxford scores between both treatment groups. Additionally, using Mersilene tape for CC suture fixation may reduce the need for secondary surgical intervention for implant removal, without the need for secondary admission or further medical expenditures.

## Abbreviations

AC: Acromioclavicular
CC: Coracoclavicular
UCLA: University of California at Los Angles
